# Bacterial contamination of medical face mask wearing duration and the optimal wearing time

**DOI:** 10.3389/fcimb.2023.1231248

**Published:** 2023-10-02

**Authors:** Guotao Ding, Guiying Li, Mengyu Liu, Peng Sun, Danqi Ren, Yan Zhao, Teng Gao, Guoxing Yang, Yanfei Fang, Weihao Li

**Affiliations:** ^1^Microbiota Division, Handan Municipal Centre for Disease Control and Prevention, Handan, Hebei, China; ^2^Urology Depart, Affiliated Hospital of Hebei University of Engineering, Handan, Hebei, China; ^3^Department of Anesthesiology, Handan Central Hospital, Handan, Hebei, China

**Keywords:** medical mask, MALDI-TOF, 16SrRNA gene sequencing, bacteria contamination, wearing time​

## Abstract

**Introduction:**

Bacterial contamination is a critical parameter for how long a medical mask will be worn.

**Methods:**

In this study, we used the pour plate method to observe the total bacteria counts in used medical face masks. The bacterial community analysis was detected using bio-Mass spectrometry technology and 16SrRNA gene sequencing technology. The wearing time of the mask from 0.5 hours to 5 hours were studied.

**Results:**

These results shown that the total number of bacteria on the inside surface of the mask were higher than the outside. The total number of bacteria on the inner surface of masks worn for 0.5 h, 1 h 2 h, 4 h and 5 h was 69 CFU/m2,91.3 CFU/m2, 159.6 CFU/m2, 219 CFU/m2, and 879 CFU/m2, respectively. The total number of bacteria on the outside surface of masks worn for 0.5 h, 1 h 2 h, 4 h and 5 h was 60 CFU/m2, 82.7 CFU/m2, 119.8 CFU/m2, 200 CFU/m2, and 498 CFU/m2, respectively. The bacterial abundance obtained from bio-Mass spectrometry were consistent with the results of 16SrRNA sequencing. Both the methods discovered the maximum number of *Neisseria* followed by *Corynebacterium* species in mask worn 5 hours. The top 100 bacteria isolated from inside and outside surface of mask belong to 11 phyla.

**Conclusions:**

We analyzed bacterial penetration efficiency of the bacteria that were detected both on the inside and outside surface of the masks. In the top 10 bacteria, no bacteria were detected both inside and outside the mask worn for four hours, while 6 bacteria species were detected on the inside and outside of the mask after wearing for five hours. Bacterial penetration rates ranged from 0.74% to 99.66% for masks worn continuously for five hours, and the penetration rate of four strains exceeded 10% in the top 10 colonies. We recommend timely replacement of masks worn for more than four hours.

## Introduction

A global pandemic caused by the novel coronavirus (COVID-19) was declared by the World Health Organization (WHO) on March 11, 2020 (Cucinotta and Vanelli, 2020). The respiratory disease has heightened the awareness of wearing the medical face mask in public place and work place. Wearing masks can restrict the spread of the virus through aerosol in the air (Brienen et al., 2010; Han et al., 2021). The global consumption of face masks has skyrocketed under the COVID-19 pandemic, and some researchers report that 129 billion face masks were consumed per month (Prata et al., 2020). Each mask contains approximately 4.5 grams of polymeric materials, and discarded facemasks should be disposed as medical waste not be recycled waste (Crisafi et al., 2022). So mass-produced and discarded medical masks will result in environmental pollution, resource consumption and other problems (Bernardo et al., 2014; Prata et al., 2020; Sills and Adyel, 2020). In this study, we research the bacterial contamination of wearing medical masks in the office environment, and recommend optimal wearing time. We aim to reduce the environmental impact of mask disposal by reducing the waste of masks while ensuring their protective efficiency.

Bacterial contamination is a critical parameter for how long a medical mask will be worn. In this study, we used the pour plate method to observe the total bacteria counts in used medical face masks (Terrones-Fernandez et al., 2023). Pour plate method was developed for the microbiological enumeration of food and pharmaceutical products (Alang et al., 2019; Humudat et al., 2020). We use the total number of bacteria as a basis to judge the degree of bacterial contamination. At the same time, we use the Matrix-Assisted Laser Desorption/Ionization Time of Flight Mass Spectrometry (MALDI-TOF-MS) to identify each single colony on the plate (Westblade et al., 2015).

The MALDI-TOF MS is an efficient technique which can measure the exact size of peptides and small proteins and build protein microbial fingerprinting (PMF) (Giebel et al., 2010). We can measure the PMF of bacteria by MALDI-TOF MS and match with the PMF library to achieve bacterial species identification. The newest Biotyper (Bruker) library contains 704 genera which include 4274 unique bacterial species. The MALDI-TOF MS can also identify the unknown bacteria by the masses of peptides of the unknown organisms’ biomarkers (Singhal et al., 2015). This technology can identify microorganism including bacterium, fungus, and parasites (Suranyi et al., 2023; Torrico et al., 2023). In this research, we used MALDI-TOF MS technique to identify single colonies isolated from masks worn for 4 and 5 hours. The results of bacterial identification acquired by MALDI-TOF MS were based on proteomic analysis. Also, the 16SrRNA sequencing technique was used for the bacterial community analysis.

16S rRNA sequencing is a method for classify bacteria based on the discrepancies of the 16S rRNA gene sequences. The V3 and V4 hypervariable regions of the 16S rRNA gene were the general targets for researching the bacterial ecology (Fadrosh et al., 2014). 16S rRNA Amplicon Sequencing was widely used in microbial communities including food, surface water and groundwater (Brandt and Albertsen, 2018; Bloomfield et al., 2023; Lyons et al., 2023). We believe that bacteria detected both on the inside and outside of the mask are those that penetrate the mask. In this context, we defined the bacterial penetration rates as: Among the top 10 identified bacteria at species levels, the bacteria detected both inside and outside the mask were selected, and the ratio was divided by the bacteria with small relative abundance by the bacteria with large relative abundance, regardless of whether the inner surface had high relative abundance or the outer surface had high relative abundance. We recruited 14 healthy volunteers to wear medical masks in a daily office environment. In this study, the office environment refers to a civilian office environment with a temperature of 15°C-35°C and a relative humidity between 30% and 65%, and no obvious dust pollution. The daily office environment chosen for this research can bring the results of this study in line with the real situation of the majority of citizens wearing masks. The bacterial penetration rates of masks with different wearing periods were compared, and the recommended wearing time of masks was finally obtained.

## Materials and methods

### Chemicals and instrument

In this study, the medical masks were produced by Wenner company (China), which is sterilization, synthetic blood no penetration under16Kpa, bacterial filtration efficiency under 95%, and particle (The diameter is 0.075 ± 0.02 μm) filtration efficiency under 30%. Nutrient agar (NA) is purchased from hepobio (Shandong province, China). Phosphate-Buffered dilution water is obtained from Land Bridge (Beijing, China). Bacterial Test Standard (BTS) (Mass Pure Grade), and Matrix alpha-cyano-4-hydroxycinnamic acid (HCCA) (Mass Pure Grade) for MALDI-TOF-MS are purchased from Bruker (Germany). Formic acid (FA) (High performance liquid chromatography, HPLC grade), Standard solvent (SS) (Mass Pure Grade) contained pure water 475 μL, trifluoroacetic acid (TFA) 25 μL, and acetonitrile 500 μL are obtained from HoneyWell (Germany).

Biological safety cabinet used in this research is purchased from AirTECH (Jiangsu province, China). Microbiological incubator is obtained from Zhicheng (Shanghai, China). Bruker MALDI Biotyper used in this study is purchased from Bruker (Germany). 16SrRNA sequencing service is provided by the NOVOGene Co. (Beijing, China).

### Mask-wearing plan

To investigate bacterial contamination on worn medical masks in an office environment, we recruited 14 volunteers to wear masks during work hours, instead of using a simulator. The volunteers ranged in age from 21 to 48 (average age 32), with seven women and seven men. All the volunteers were free of oral, facial skin, and respiration diseases. Each person wore a mask once a day, the wearing time was 0.5, 1, 2, 4, 5 hours, respectively, and each wearing time was repeated 3 times. In this research, 210 masks were used for the study. At the end of the wearing time, the mask was cut into pieces in a biological safety cabinet, and the pieces on the inside surface and outside surface were stored in a sterilized centrifuge tube at -20°C.

### Isolation of bacteria

The edge of the mask was cut, The inside and the outside separated (the spread area is about 12 centimeters ×10 centimeters), cut it into pieces, and these pieces were put into 50ml centrifugal tube with 20 ml Phosphate-Buffered dilution water. A tenfold serial dilution was performed by adding 1ml of sample extraction solution to a dilution tube containing 9ml of Phosphate-Buffered dilution water. The dilution is up to 10^-3^ of the original extraction solution.

The concentration of the NA is 33 g/L (containing protein peptone 10 g/L, beef peptone 3 g/L, sodium chloride 5 g/L, and agar 15 g/L.). The medium was sterilized by autoclave at 121°C for 15 mins and then transferred to a 48°C water bath on the same day for the experiment (Sanders, 2012). Take 1 ml of serial dilution of three concentrations of 10^-1^, 10^-2^, and 10^-3^ respectively into an empty Petri dish, and pour into the medium of 20ml medium at 48°C. Three replicates per concentration. Gently swirl the plate for mixing he sample with the agar. After the agar thoroughly solidify, invert the plate for incubation at 36°C ± 1 for 48 hours. The total number of bacteria was counted, multiplied by the dilution factor, divided by 120 cm^2^(12cm×10cm), and the final to CFU/cm^2^.

### MALDI-TOF-MS

In this study, we use the extended direct transfer procedure to identify the isolates. This procedure consists of 3 steps. First, smear one fresh colony isolated on the MADLI target plate, and allow it to dry at room temperature. Second, add 1μL 70% formic acid in water to the dried chip. Last, cover 1 μL matrix HCCA on the chip. After the above steps are completed, the MADLI target plate can be detected on the MALDI Biotyper.

Before testing, the MALDI-TOF-MS has to be tuned using BTS. ​The spots covered by the BTS had to be detected 6 times and the signals superimposed. Observe whether the peak shape of mass-to-charge ratio (m/z) 16952 and 13683 were sharp and symmetrical. If the peak is good enough, we can assign the calibration point of BTS and apply the method for bacterial identification. ​The results obtained from MALDI-TOF-MS will be matched with the commercial library (the MALDI Biotyper software 3.0) from Bruker Daltonik (Pandey et al., 2019). If the score values of the matching results range from 2.0 to 3.0, the results will be available for bacterial identification.

### 16SrRNA sequence

In this research, worn medical masks were sent to Novogene biotechnology Ltd. Using the SDS (sodium dodecyl sulfate) method extracted the genomic DNA. Add 0.5 ml 10% SDS, 0.4 M EDTA, 50 mM Tris (pH 8.0), 1 mg/ml proteinase K. Incubate in 50°C water bath for 2 hours. Extract with 800μL chloroform and centrifuge at 12000 rpm for 15 min. Transfer supernatant to new tube. Add 500μL Isopropanol to the tube, and put it in the refrigerator at -20°C for 30 mins. The top layer was removed by centrifugation at 12,000 rpm for 5 min. Spool out DNA and dissolve in 200 μl of pure water. The sequencing targets were the V3 and V4 hypervariable regions of the 16S rRNA gene which were amplified with barcoded primers 341F (5’-CCTAYGGGRBGCASCAG-3’) and 806R (5’-GGACTACNNGGGTATCTAAT-3’). The NEBNext^®^ Ultra™ IIDNA Library Prep Kit was used for library preparation. The Qubit method is a technique for accurately quantifying DNA using Qubit™ 2.0 Fluorometer (Thermo Fisher Scientific Inc., American). The Qubit method and the Q-PCR (Quantitative Real-time-PCR) method were applied to test whether the constructed library is qualified. The Qubit method was performed by using Qubit^®^ dsDNA HS Assay Kits. Add 100μL the DNA for Library preparation and 100μL Qubit dsDNA HS Master Mix to a tube. It was placed at room temperature for 3 mins and tested on the Qubit^®^ 2.0 Fluorometer (Thermo Fisher, American). The Q-PCR method was performed by KAPA Library Quantification Kit. Add 12μL the KAPA SYBR FAST qPCR Master Mix, 4μL double distilled water, and 4μL DNA for Library preparation. The q-PCR process consisted 3step: step one pre-denaturation at 95°C for 5 mins, step two denaturation at 95°C for 30 seconds, and step three annealing/extension at 60°C for 45 seconds. 35 cycles for step two and three. A DNA concentration of more than 0.2 ng/μL can be used for gene sequencing. The library sequencing was performed on the NovaSeq 6000 Sequencing System, and 250 bp paired-end reads were generated.

### 16S bioinformatics analysis

After removing the primer sequence, the FLASH (Fast Length Adjustment of SHort reads) software (Version 1.2.11, http://ccb.jhu.edu/software/FLASH/) was applied to splice the reads to get raw tags. Process the raw tags using Fastp (Version 0.20.0) software to obtain clean tags. Vsearch software (Version 2.15.0) is used to compare clean tags with the database to get the effective tags. To get ASVs (Amplicon Sequence Variants) (default: DADA2), effective tags were filtered by the DADA2 module of QIIME2 software (Version QIIME2-202006). We deleted all OTUs containing <5 sequences for quality control. The resulting ASVs are then compared with the Silva138.1 database using the classify-sklearn module in QIIME2 software to obtain species information for each ASV.

### Statistical analysis

CFU (Colony-FormingUnits) is the mean of each series wearing duration of 3 masks. We applied the Wilcoxon test to analyze the number of total bacteria with wearing time (0.5h, 1h, 2h, 4h, and 5h). The identification results obtained from MALDI-TOF-MS were repeated three times. In order to analyze the diversity, richness and uniformity of the communities in the sample, alpha diversity was calculated from 7 indices in QIIME2 (Version QIIME2-202006), including Observed_otus, Chao1, Shannon, Simpson, Dominance, Good’s coverage and Pielou_e.

## Results and discussion

### Results of total bacteria

**Figure 1:**
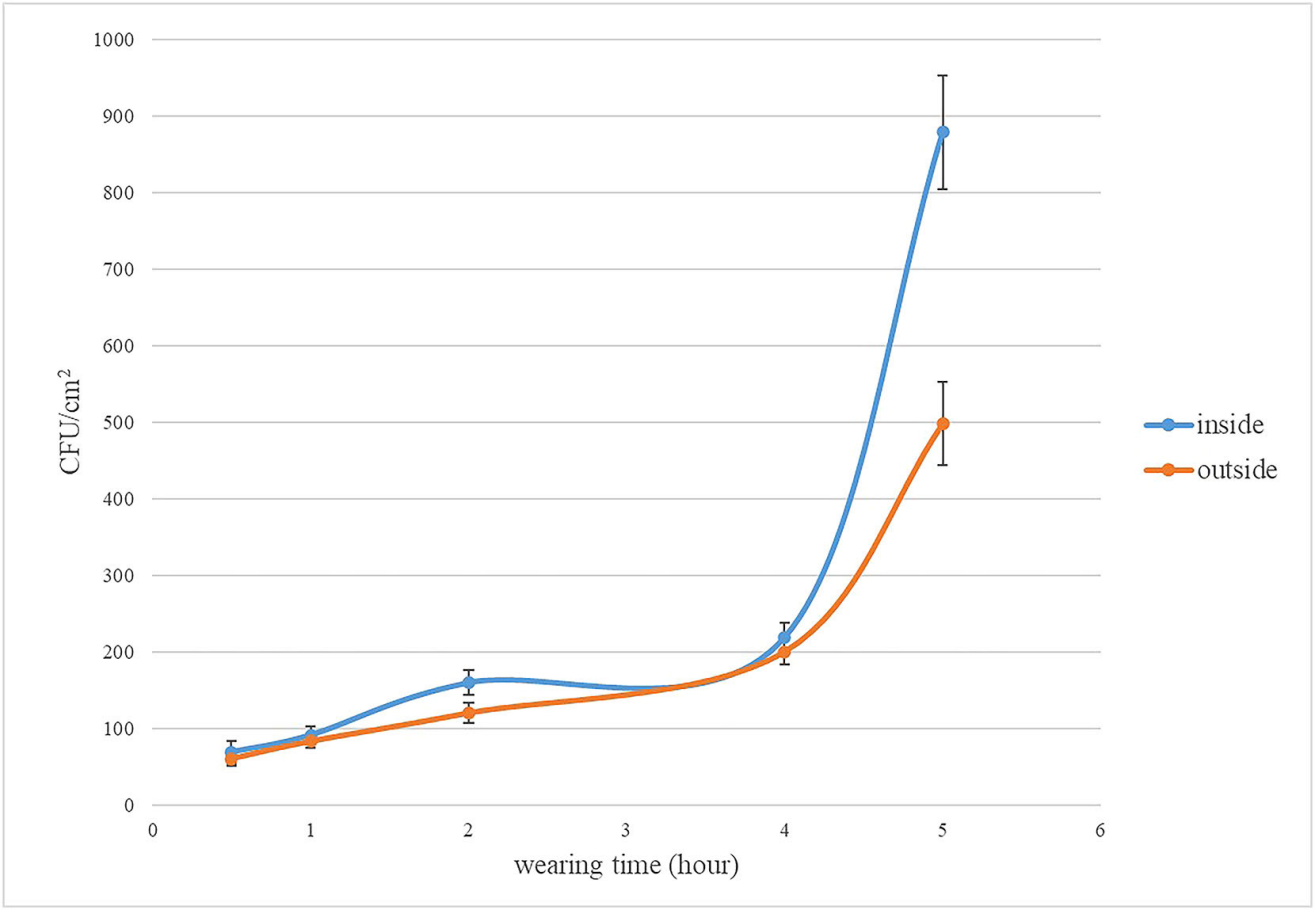
The total number of bacteria on the inside and outside of mask. The error line of the bar chart was the sample standard deviation (SD).

In three dilutions (10^-1^, 10^-2^, and 10^-3^), the plate, CFU between 30 to 300, was counted for the number of total bacteria. Below 30 CFU and above 300 CFU would be excluded from the mean of three duplication in each dilution. If all dilutions are less than 30CFU, the total number of bacteria will be calculated by the number of colonies in the least dilute plate. The results are illustrated in **Figure 1**. The total number bacteria on inside surface were higher than the outside. The total numbers of bacteria range from 69 to 219 CFU/cm^2^ (inside surface of medical face mask), and range from 60 to 200 CFU/cm^2^ (outside surface of medical face mask) wearing time from 0.5h to 4h. The total numbers of bacteria reach 879 CFU/cm^2^ (inside surface of medical face mask), and reach 498 CFU/cm^2^ (outside surface of medical face mask) wearing for 5h. The rising tendency of the total number of bacteria was relatively flat. After wearing the mask for 4 hours, a surge in the total bacteria counts was observed.

### Results of MALDI-TOF MS Identification

It is possible to identify bacteria at species level using MALDI-TOF MS. Therefore, MALDI-TOF MS is used as the reference method for 16SrRNA sequencing in the bacterial community. In this research, 325 colonies, which belong to all wearing periods from 0.5h to 5h, were identified using MALDI-TOF MS, among which the unassigned 17 colonies accounted for 5.2%. The top 5 bacterium identified by MALDI-TOF MS were *Neisseria oralis* 24 (7.3%), *Fusobacterium periodonticum* 20 (6.2%), *Corynebacterium pyruviciproducens* 19 (5.8%), *Corynebacterium tuberculostearicum* 16 (4.9%), and *Haemophilus parainfluenzae* 13 (4.0%) (**Table 1**). The score value ranging from 2.00 to 3.00 is mean for high confidence identification. In this study, all score value of the bacterium identified by the MALDI-TOF MS were above 2.00.

**Table 1:** Identification results of bacterium on the worn medical mask using MALDI-TOF MS.

Microorganism	G+/G-	No. of strains	Percents in the bacterial community (%)
*Neisseria oralis*	G**-**	24	7.3
*Fusobacterium periodonticum*	G**-**	20	6.2
*Corynebacterium pyruviciproducens*	G+	19	5.8
*Corynebacterium tuberculostearicum*	G+	16	4.9
*Haemophilus parainfluenzae*	G**-**	13	4.0
*Schaalia odontolytica*	G+	11	3.4
*Rothia aeria*	G+	8	2.5
*Streptococcus sanguinis*	G+	6	1.8
*Acinetobacter schindleri*	G**-**	6	1.8
*Corynebacterium durum*	G+	4	1.2
others	–	181	55.9
unassigned	–	17	5.2

G+ is gram-positive bacteria. G- is gram-negative bacteria. – is none.

### Results of 16SrRNA sequence

#### The results of bacterial species

**Figure 2:**
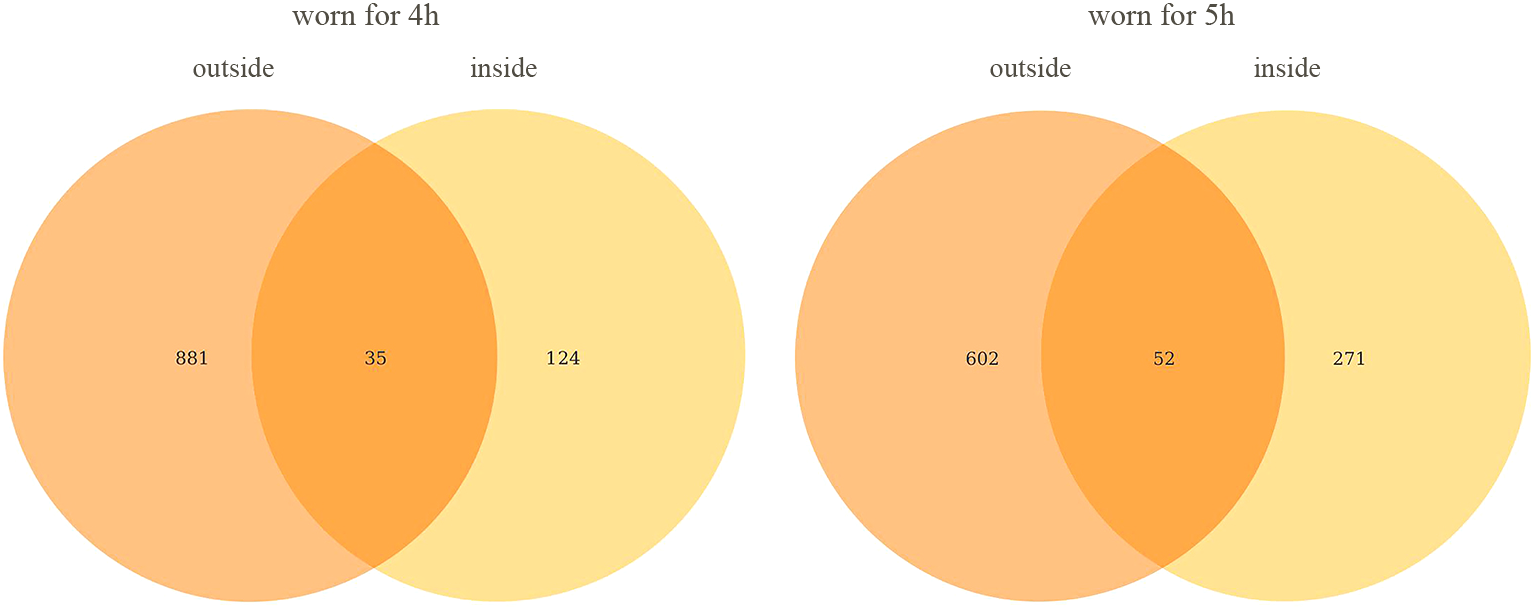
Venn diagram of ASVs on the inside and outside of mask. All data in the figure represent the number of ASVs and not their abundance.

​The 16S sequencing technique was applied to masks worn for four hours and five hours for bacterial communities. Outside surface of masks worn for four hours contain 881ASVs, inside surface of masks worn for four hours contain 124 ASVs, and 35 ASVs were detected both inside and outside. Outside surface of masks worn for five hours contain 602 ASVs, inside surface of masks worn for five hours contain 271 ASVs, and 52 ASVs were detected both inside and outside. The results were shown in **Figure 2**. The results showed that the longer the mask was worn, the more types of bacteria were detected both inside and outside the mask.

In the process of data analysis, we found a very interesting phenomenon that the total number of bacteria on the outside surface of the mask was lower than that on the inside surface, but the number of bacterial species was significantly higher than that on the inside surface. We try to give a reasonable explanation for this phenomenon. In this research, we recruit volunteers for the study, rather than using a simulator. The bacteria on the outside surface is from the air and environment, and the bacteria on the inside surface is from the oral and face. On the inside surface of the mask, the oil secreted by the skin, the water vapor exhaled and the saliva can all serve as the nutrients of the bacteria, so the total number of bacteria is higher than that on the outside surface. The bacteria in the air and environment are more diverse than those in the mouth and face, so the outside surface has a higher variety of bacteria than the inside surface.

#### The relative abundance for top 10 bacteria

**Figure 3:**
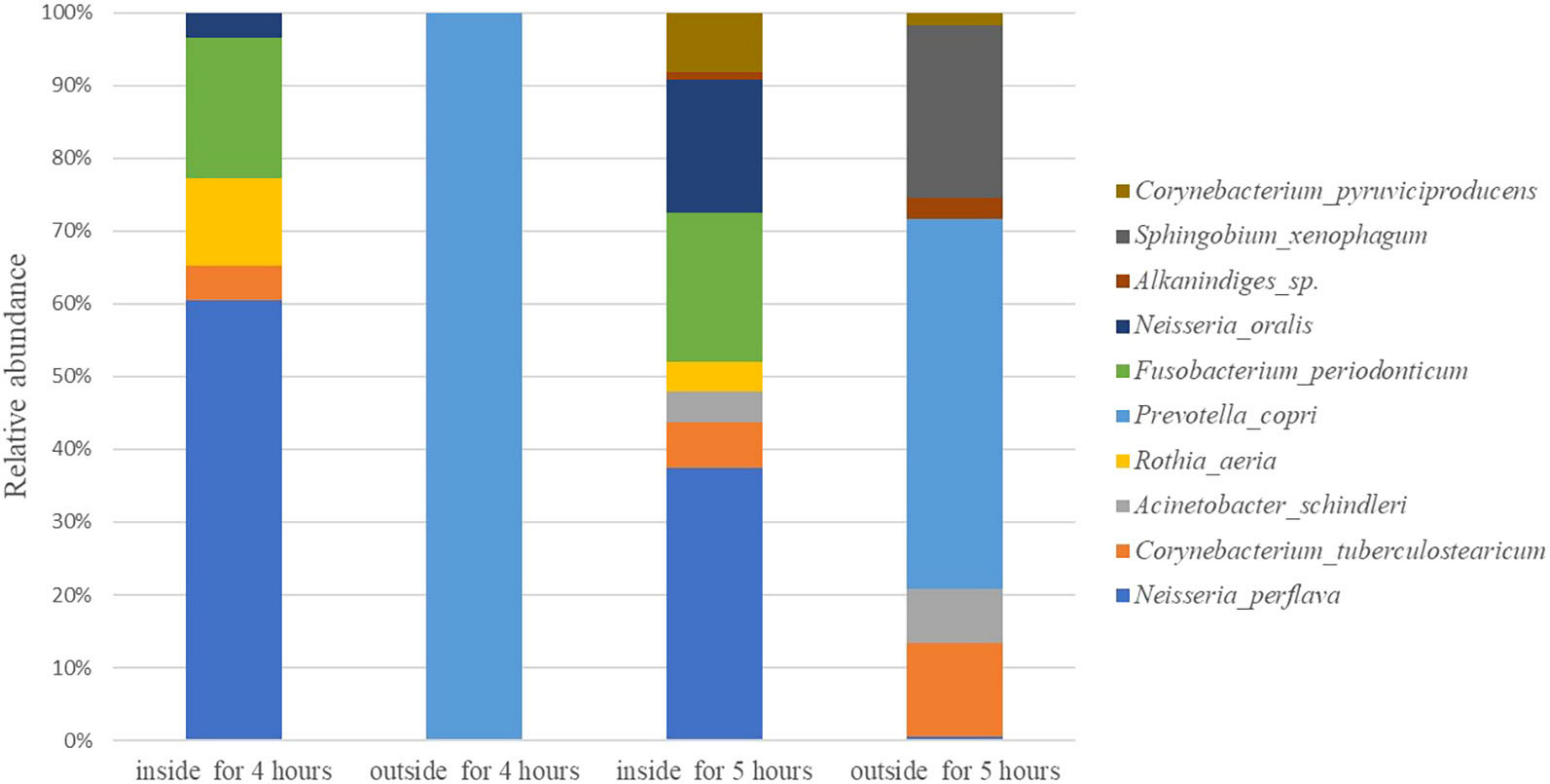
The relative abundance of Top 10 bacteria on the worn mask.

In this paper, we analysis the all bacteria identified at species level. Rank the maximum relative abundance of bacteria at identified species levels in each sample. The top ten are shown in **Figure 3**. The top 10 bacteria are *neisseria perflava, Corynebacterium tuberculostearicum, Acinetobacter schindleri, Rothia aeria, Prevotella copr, Fusobacterium periodonticum, Neisseria oralis, Alkanindiges* sp.*, Sphingobium xenophagum, and Corynebacterium pyruviciproducens*. The results of the top 10 species obtained from 16SrRNA sequence are consistent with those obtained from MALDI-TOF MS.

**Figure 4:**
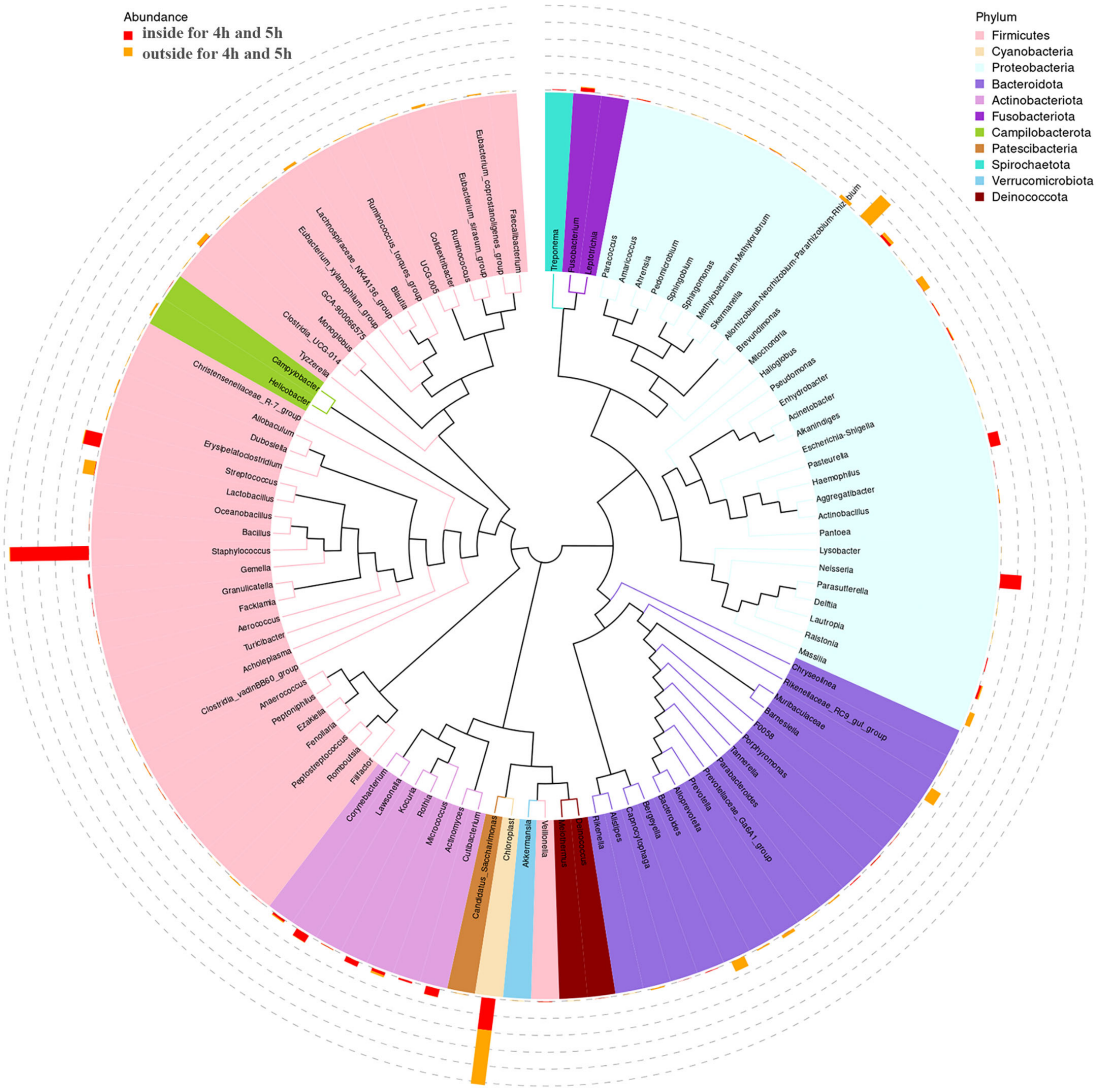
The tree of phylogenetic diversity in worn medical face masks. Branches and fan colors representing their corresponding phyla, and stacked bar graphs outside the fan ring representing the abundance distribution information of the genus in different samples.

The phylogenetic diversity of bacteria isolated from inside and outside surface of mask was shown in **Figure 4**. The top 100 ASVs belong to 11 phyla. The top 11 phyla are *Firmicutes, Cyanobacteria, Proteobacteria, Bacteroidota, Actinobacteriota, Fusobacteriota, Campilobacterota, Patescibacteria, Spirochaetota, Verrucomicrobiota, and Deinococcota.*

### Results of bacterial penetration rates

In the top 10 bacteria, no bacteria were detected both inside and outside the mask worn for four hours, while 6 bacteria species were detected on the inside and outside of the mask after wearing for five hours. The bacterial penetration rate of *Corynebacterium tuberculostearicum* is 99.66%, *Acinetobacter schindleri* is 86.29%, *Alkanindiges* sp. is 80.30%, *Corynebacterium pyruviciproducens* is 10.47%, *Rothia aeria* is 1.04%, and *Neisseria perflava* is 0.74%. The results were shown in **Table 2**.

**Table 2:** The results of bacterial penetration rates for masks worn 5 hours in top 10 bacteria.

Microorganism	Relative abundance (%)		penetration rates
	inside for 5 hours	outside for 5 hours	
*Neisseria perflava*	2.9681871	0.0219866	0.74%
*Corynebacterium tuberculostearicum*	0.4972348	0.4955435	99.66%
*Acinetobacter schindleri*	0.3331811	0.2875167	86.29%
*Rothia aeria*	0.3247247	3.38E-03	1.04%
*Prevotella copri*	0	1.9618787	–
*Fusobacterium periodonticum*	1.6337714	0	–
*Neisseria oralis*	1.4426573	0	–
*Alkanindiges* sp.	0.0896376	0.1116241	80.30%
*Sphingobium xenophagum*	0	0.9183622	–
*Corynebacterium pyruviciproducens*	0.6460669	0.067651	10.47%

– is none.

### The optimal wearing time for medical face mask

Before this study, some researchers invested in the bacterial filtration efficiency (BFE) and breathability of the medical face masks, and recommended the optimum of wearing time for masks being 8 hours (Armand et al., 2022). This argument is very persuasive, but it has some shortcomings. First, the study used simulators without using volunteers to wear masks, which may result in unreal wearing parameters. Second, the study did not consider bacterial buildup and contamination after normal mask wear. Some studies have shown that bacteria attached to masks can survive for up to eight hours (Huang et al., 2020). For this reason, we analyze total bacteria, bacterial communities, and bacterial penetration rates. The inflection point at which the total number of bacteria increased was taken as one parameter, after which the total number of bacteria increased sharply, and the bacterial penetration rate was taken as another parameter to determine the optimal wearing time of the mask.

After wearing the mask for a long time, skin pores and wrinkles showed significant increases (Park et al., 2021). Mechanical integrity was not affected by folding, ageing, and washing masks (Varanges et al., 2022). Some colleagues have revealed that filtration efficiencies (FEs) sharply decreased with the increasing of wearing time for masks (Guan et al., 2022; Li et al., 2022). Overall, we recommend timely replacement of masks worn for more than four hours.

## Conclusions

According to the total bacterial count and bacterial penetration rate, we recommend changing masks every 4 hours if conditions permit. We research the bacterial contamination of worn medical face masks using volunteers wearing mask rather than a simulator. Using this method, we can get the real data for bacterial contamination. The total number of bacteria on both the inside and outside surfaces of the mask increased dramatically after more than 4 hours. The top 10 bacteria identified by MADLI-TOF were consistent with the top 10 bacteria identified by 16srRNA sequencing, which also proved the credibility of the genetic sequencing results. Based on the 16srRNA sequencing results, the phenomenon of bacteria penetrating the mask appeared in the top 10 bacteria only after wearing the mask for more than 4 hours. The penetration rate of four strains exceeded 10% in the top 10 colonies for masks worn continuously for five hours.

## Data availability statement

The data presented in the study are deposited in the figshare repository, accession DOI. is DOI.10.6084/m9.figshare.24174093.

## Author contributions

GD: Conceptualization, Methodology, Writing- Original draft preparation. GL and ML: Software. PS and DR: Data curation. YZ and TG: Visualization, Investigation. GY and YF: Supervision. WL: Writing- Reviewing and Editing. All authors contributed to the article and approved the submitted version.
